# Application of Single-Atom Nanozymes in the Detection of Small Biomolecules: A Review

**DOI:** 10.3390/molecules31081242

**Published:** 2026-04-09

**Authors:** Wanyi Zhang, Rong Huang, Wenhui Luo, Xiaojing Si, Dongmei Deng, Liqiang Luo

**Affiliations:** 1 College of Sciences, Shanghai University, Shanghai 200444, China; zwyshu2023@shu.edu.cn (W.Z.); 22820048@shu.edu.cn (R.H.); wenhui@shu.edu.cn (W.L.); dmdeng@shu.edu.cn (D.D.); 2 Department of Food Science and Nutrition, Shanghai Business School, Shanghai 200235, China

**Keywords:** single-atom nanozymes, small biomolecules, detection

## Abstract

Single-atom nanozymes (SANs) with atomically dispersed metal sites show great potential in small biomolecule detection. This review first summarizes SAN synthesis (wet chemistry, atomic layer deposition, etc.), structural features (tunable coordination, metal-carrier interactions), and catalytic mechanisms (synergistic effects, d-band modulation). Afterwards, this review focuses on the applications of SANs in detecting small biomolecules, including glucose, glutathione, uric acid, ascorbic acid, hydrogen peroxide, and dopamine via colorimetry, fluorescence, and electrochemistry. Challenges such as matrix interference and stability, along with future directions in flexible electronics and clinical translation, are discussed, aiming to advance SAN-based detection technologies.

## 1. Introduction

Small biomolecules (such as metabolites, neurotransmitters and antioxidants) exhibit characteristic concentration shifts or spatiotemporal distribution differences during disease development [1,2]. Precise detection of these small biomolecules provides critical data support for studying disease pathological mechanisms and clinical diagnosis and treatment. For instance, tumor cells show significantly elevated extracellular hydrogen peroxide (H_2_O_2_) concentrations due to the Warburg effect, reaching 3–5 times as high as that of normal tissues. Monitoring H_2_O_2_ levels in the tumor microenvironment enables real-time tracking of cancer cell proliferative activity and evaluation of radiotherapy/chemotherapy sensitivity [3]. In diabetic patients, blood glucose concentrations persistently exceed the normal threshold (fasting blood glucose ≥ 7.0 mM), and dynamic monitoring of blood glucose fluctuation curves directly informs insulin dosage adjustment in treatment regimens [4]. Additionally, cerebrospinal fluid dopamine (DA) levels in neurodegenerative disease patients decrease significantly (by 60%–80%), with concurrent reductions in its metabolite homovanillic acid, serving as important biochemical markers for early Parkinson’s disease diagnosis [5]. In patients with chronic kidney disease, levels of circulating uric acid (UA) and creatinine rise progressively, with UA exceeding 420 μM or a reduced creatinine clearance below 60 mL/min/1.73 m^2^ serving as quantitative markers for renal impairment [6,7]. In oxidative stress-related diseases, intracellular glutathione (GSH) and cysteine (Cys) concentration imbalances are particularly pronounced. As a major antioxidant, decreased GSH content weakens cellular antioxidant capacity, while abnormal Cys accumulation, which is critical for protein disulfide bond formation, triggers protein misfolding. Dynamic monitoring of both molecules helps reveal disease oxidative damage mechanisms and guides the formulation of antioxidant therapeutic strategies [8,9].

Current mainstream detection technologies for small biomolecules, such as high-performance liquid chromatography [10], gas chromatography–mass spectrometry [11], and enzyme-linked immunosorbent assay [12], demonstrate certain advantages in sensitivity and specificity, but still have significant technical bottlenecks. However, these techniques are often constrained by high analytical costs and complex, time-consuming sample pretreatment, making them unsuitable for rapid on-site monitoring [13]. Although enzyme-linked immunosorbent assay technology has the characteristic of relatively simple operation, it relies on the preparation of specific antibodies. Nonetheless, antibody production remains limited by long development cycles and high manufacturing costs. In addition, this method has the problem of cross-reactivity and is easily interfered with by matrix effects in complex biological samples, resulting in a decrease in the accuracy of detection results [14]. The drawbacks of conventional approaches in terms of throughput, cost efficiency, and real-time on-site analysis highlight the urgent need for novel detection strategies to enable rapid and low-cost monitoring of small biomolecules in clinical diagnostics, environmental surveillance, and related applications.

Nanozymes are a class of nanomaterials with enzyme-mimetic catalytic activity, and the proposal of this concept has broken the traditional perception that enzymes are solely composed of proteins or nucleic acids [15,16]. In 2007, a team reported for the first time that Fe_3_O_4_ nanoparticles exhibit peroxidase (POD)-like activity, catalyzing the colorimetric reaction between 3,3′,5,5′-tetramethylbenzidine (TMB) and H_2_O_2_, a finding that effectively inaugurated nanozyme research [17]. Subsequently, metal nanozymes [18,19], metal oxide nanozymes (MnO_2_, CeO_2_) [20,21], and carbon-based nanozymes (graphene quantum dots, and carbon nanotubes) [22,23] have been successively developed. Through the precise modulation of nanomaterial dimensions, structural morphology, and surface chemistry, researchers have successfully mimicked the catalytic behaviors of various natural enzymes, such as oxidoreductases, hydrolases, and oxidases (OXDs). However, traditional nanozymes have issues such as ambiguous active sites, limited catalytic efficiency, and insufficient substrate selectivity, which restrict their in-depth application in the field of bioanalysis [24,25].

The emergence of single-atom nanozymes (SANs) has provided a breakthrough solution to the aforementioned challenges. Precisely anchoring individual metal atoms as isolated sites on supports enables, in principle, near-unity atomic utilization and avoids the catalytic inactivity associated with the bulk atoms in conventional nanoparticles [26,27]. With the aid of characterization techniques such as high-angle annular dark field scanning transmission electron microscopy (HAADF-STEM) and X-ray absorption fine structure spectroscopy (XAFS), studies have confirmed that the active centers of SANs have well-defined coordination structures (M-N_4_, M-O_4_) [28]. Through regulating the metal type, coordination environment, and carrier properties, the precise adjustment of electron cloud density and spin state can be realized, thereby significantly improving catalytic activity and substrate specificity. For example, Co-N-C SANs display catalytic activity in the H_2_O_2_-driven colorimetric reaction that is roughly 100-fold higher than that of conventional Co nanoparticles, while their selectivity for TMB is increased by about three times [29]. Moreover, intense metal–support interactions that anchor single atoms to the support impart excellent chemical stability, allowing the material to retain its structural integrity and catalytic performance under high temperature, extreme pH, or in complex biological matrices, thus avoiding the denaturation and inactivation that commonly affect natural enzymes [30,31]. Owing to these features, SANs are particularly advantageous for detecting small biomolecules: their high atom utilization, together with adjustable catalytic properties, enables strong amplification of assay signals. By designing coupling systems of specific recognition elements (such as aptamers and antibodies) and SANs, highly sensitive colorimetric, fluorescent, or electrochemical sensing platforms can be constructed [32,33]. Moreover, the excellent stability of these systems provides strong resistance against interference from complex biological matrices (e.g., serum, urine, and cell lysates), allowing for precise quantification of analytes, including glucose (a diabetes biomarker), ascorbic acid (AA), GSH (tumor-related metabolites), and neurotransmitters, such as DA and serotonin [34,35]. This approach is anticipated to overcome the constraints of conventional detection techniques regarding analytical sensitivity, selectivity, and throughput [36,37,38].

## 2. Synthesis, Characterization, and Structure of Single-Atom Nanozymes

The design and synthesis of SANs serve as the foundation for achieving their efficient catalytic performance and precise detection applications. Through rational regulation of the coordination environment of metal atoms [39], structural characteristics of carrier materials, and synthesis process parameters [40,41], the active site distribution, electronic structure, and stability of SANs can be directionally optimized [42]. These findings provide a structural framework for interpreting the catalytic mechanisms and supply essential technical foundations for the design of highly sensitive, selective sensing platforms for small-molecule analysis.

### 2.1. Synthetic Methods

The controllable synthesis of SANs is a prerequisite for optimizing their catalytic performance and enabling detection applications. Various synthesis methods, each founded on distinct physicochemical principles, have been developed to date. These approaches enable rational design of SANs by permitting precise control of metal-atom dispersion, metal–support interfacial interactions, and the coordination environment of active sites. Among them, mainstream techniques possess unique advantages and suitable application scenarios, including wet chemical methods, atomic layer deposition (ALD), high-temperature pyrolysis, and template-assisted methods [40,43].

**Figure 1:**
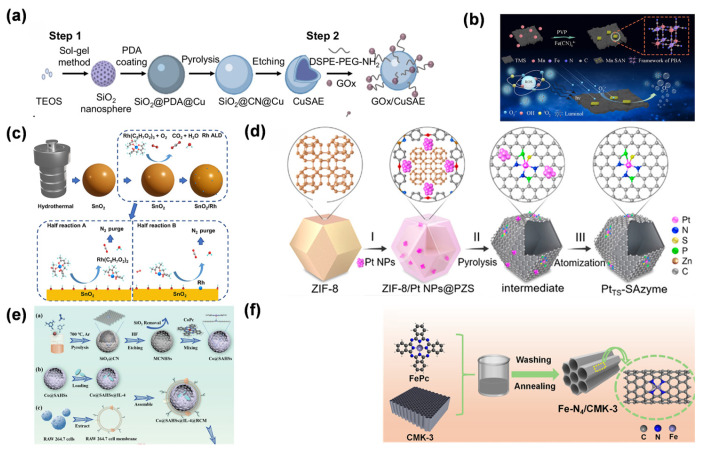
(**a**) Disulfidptosis induced by GOx/Cu SAN [44]. Copyright (2023) Spring Nature. (**b**) Mn-SAN prepared by co-precipitation [45]. Copyright (2023) American Chemical Society. (**c**) Synthesis and gas sensing of Rh-SAN/SnO_2_ [48]. Copyright (2023) Elsevier. (**d**) Synthesis and characterization of thermally stable Pt SAN [43]. Copyright (2021) American Chemical Society. (**e**) Synthetic process of Co SAN [51]. For this subfigure, a represents the schematic illustration of the synthetic process of Co@SAHSs; b and c represent the schematic illustration of the synthetic process of Co@SAHSs@IL-4@RCM. Copyright (2023) Wiley. (**f**) Synthetic process of Fe-N_4_/CMK-3 [52]. Copyright (2023) American Chemical Society. These diverse methodologies illustrate the feasibility of achieving isolated metal sites across various supports, ensuring maximized atomic utilization and structural stability for subsequent catalytic reactions.

Wet chemical methods, as a mainstream synthetic strategy for SANs, achieve controllable immobilization of metal atoms and integration with carriers through coordination chemistry and reduction reactions in solution systems. This approach introduces chelating agents (such as ethylenediaminetetraacetic acid and sodium citrate) or ligand molecules (e.g., azacyclic organic compounds), which form stable complexes by virtue of their strong complexing ability with metal ions, to reduce the activity of metal ions while inhibiting their tendency to agglomerate. Subsequently, atomic-level dispersion is achieved through reducing agents or pyrolysis. Among these, the sol–gel method forms a 3D network gel through the hydrolysis-condensation reaction of metal alkoxides, uniformly encapsulating metal ions in the carrier precursor, and obtaining a single-atom dispersed structure after calcination. In the study by Liu et al., Cu SANs were synthesized using the sol–gel method. Specifically, SiO_2_ nanosphere cores were prepared through the sol–gel process of tetraethoxysilane [44]. Subsequently, polydopamine was coated on their surface, and copper precursors were introduced. After high-temperature pyrolysis, polydopamine was converted into a carbon–nitrogen framework. Finally, the SiO_2_ cores were etched away to obtain hollow carbon–nitrogen sphere structures loaded with Cu single atoms, where Cu atoms were stably dispersed in the form of a Cu-N_4_ coordination (Figure 1a). The co-precipitation method is based on the synchronous precipitation of metal salts and carrier raw materials in alkaline solutions, anchoring metal atoms at carrier lattice defects or surface active sites by utilizing surface charge matching or lattice matching effects. In the Fu group’s work, Mn SANs were embedded into a Prussian blue analog framework assembled on Ti_3_C_2_ MXene sheets using a surfactant-assisted co-precipitation approach conducted at room temperature [45]. This approach achieved a high loading capacity of 13.5 wt%, with Mn atoms presenting a monodispersed state and exhibiting superoxide dismutase-like activity (Figure 1b). While scalable and cost-effective, wet chemical methods require precise kinetic control over parameters like pH and temperature to prevent atomic agglomeration and maintain support integrity.

ALD, a gas-phase self-limiting synthesis technique, exhibits precise atomic-level regulation capabilities in the preparation of SANs [46]. This approach relies on successive chemisorption steps in which metal–organic precursor species bind to the support surface. By pulsing gaseous precursors, metal species are sequentially deposited onto the support, forming a monolayer. The unique advantage of ALD technology lies in its self-limiting reaction characteristics, which enable uniform and atomically dispersed metal loading on the surface of carriers with complex morphologies (such as porous materials and nanofibers), avoiding the agglomeration phenomenon that easily occurs in traditional methods [47]. In the study by Zhou et al., ALD was employed to synthesize Rh-single-atom-modified SnO_2_ nanozymes (SnO_2_/Rh) [48]. With Rh(acac)_3_ as the precursor and O_2_ as the oxidant, uniform anchoring of Rh single atoms on the SnO_2_ surface was achieved through 25 Rh ALD cycles at 150 °C, involving alternating pulses of the precursor and oxidant combined with N_2_ purge steps, thereby forming a stable single-atom dispersed structure (Figure 1c). Despite high costs and limited scalability due to stringent vacuum requirements, ALD remains vital for constructing well-defined SAN models to accurately elucidate fundamental structure–activity relationships [49,50].

High-temperature pyrolysis primarily relies on the carbonization and reconstruction processes of metal–organic precursors (such as metal–organic frameworks, MOFs) under high-temperature conditions to construct single-atom dispersed structures. This method employs the ordered architectures generated by co-assembly of metal ions with organic ligands to act as templating scaffolds. Under the protection of inert gases (Ar, N_2_) or reducing atmospheres (H_2_/Ar), the organic ligands are carbonized to form carbon-based carriers through programmed temperature rise. Metal species become coordinated to heteroatoms (e.g., N, P) within the carbon framework and are ultimately immobilized on the support as isolated atomic sites. In the study by Chen et al. [43], the conversion of platinum nanoparticles (Pt NPs) to single atoms was achieved via high-temperature pyrolysis. Specifically, ZIF-8 was used as the carrier, and after loading Pt NPs, a polyphosphazene layer was coated on the surface. Through pyrolysis at 1050 °C under N_2_ atmosphere, Pt NPs were gradually atomized and anchored in the carbon carrier co-doped with N, P, and S, forming a thermally stable Pt SAN with a unique Pt_1_-N_3_PS coordination structure (Figure 1d). Pyrolysis temperature critically determines the product structure: at relatively low temperatures (400–600 °C), the limited mobility of metal atoms favors cluster formation, whereas excessively high temperatures (>1000 °C) lead to support collapse and promote coalescence of the metal atoms. Therefore, it is necessary to accurately optimize the pyrolysis curve through thermogravimetric analysis and differential scanning calorimetry. Chen et al. reported that pyrolyzing the ZIF-8/Pt NPs@poly(cyclotriphospazene-co-4,4′-sulfonyldiphenol) composite at 1050 °C in N_2_ led to the complete atomization of Pt nanoparticles and their homogeneous incorporation into the N, P, S-co-doped carbon support as Pt_1_-N_3_PS coordinated single-atom sites, with no detectable Pt clusters or particles. When the temperature was lower than 1050 °C, Pt nanoparticles could not be fully atomized, resulting in the presence of Pt clusters. In contrast, when the temperature exceeded 1100 °C, the destruction of the carbon carrier structure led to a reduction in coordination sites, causing Pt single atoms to reaggregate into particles [43]. In addition, the atmosphere composition significantly regulated the chemical state of metal atoms. A reducing atmosphere can promote the reduction in high-valent metal ions, while an inert atmosphere is beneficial to maintain the coordination structure of metal–heteroatoms. This method is widely used in the synthesis of M-N-C (M=Fe, Co, Ni)-type SANs due to the advantages of strong designability of precursor structures and scalable preparation. However, it has high requirements for the precise control of reaction kinetics, and in situ characterization techniques need to be combined to analyze the structural evolution mechanism during the pyrolysis process.

Template-assisted methods offer a unique pathway for the controllable synthesis of SANs by leveraging the spatial confinement effects of porous materials or biomolecules. This strategy uses mesoporous templates with ordered channels (e.g., MCM-41, SBA-15) or self-assembled biomolecules, exploiting their nanoscale pores, cavities, or selective binding sites to confine metal atoms and prevent their clustering. Zhang et al. prepared Co SAN by a template-assisted pyrolysis approach. Polymer-coated SiO_2_ nanospheres were subjected to high-temperature pyrolysis, producing a SiO_2_@CN composite that served as the precursor [51]. The SiO_2_ template was then etched away to obtain nitrogen-doped mesoporous carbon hollow spheres. Subsequently, Co-N_5_ single-atom active sites were constructed through coordination with cobalt phthalocyanine, achieving atomic-level dispersion and stable anchoring of cobalt atoms (Figure 1e). Mesoporous carbon hard templates, on the other hand, uniformly disperse metal precursors within the channels through the steric hindrance effect of nanoscale pores; after high-temperature pyrolysis or chemical reduction, the single-atom dispersed structure is retained during template removal. He et al. reported the fabrication of a SAN, Fe-N_4_/CMK-3, featuring atomically dispersed Fe-N_4_ sites, prepared through a hard-template approach employing mesoporous carbon (CMK-3) as the structural scaffold [52]. The specific process involved first preparing the CMK-3 mesoporous carbon carrier with SBA-15 as the template, then dispersing iron phthalocyanine in the carrier. After high-temperature pyrolysis, Fe atoms were anchored in the ordered nanochannels of the mesoporous carbon in the Fe-N_4_ configuration, forming a nanoreactor with a confinement effect (Figure 1f). The main advantage of template-assisted strategies is their ability to precisely control single-atom loading, spatial distribution, and coordination environment by tuning the template’s pore size, porosity, and surface chemical properties. Additionally, the introduction of biological templates endows the synthesis process with green, environmentally friendly, and biodegradable characteristics. Nonetheless, template-assisted strategies encounter problems such as residual template material and limited control over metal-template bonding; consequently, surface functionalization coupled with post-treatment steps is typically required to fine-tune SANs structure and performance.

### 2.2. Key Characterization Techniques

Multi-scale characterization techniques, including spherical aberration-corrected transmission electron microscopy, XAFS, and X-ray photoelectron spectroscopy (XPS), are essential for resolving the coordination environments, electronic states, and catalytic mechanisms of SANs, providing the structural evidence needed to understand their structure-activity relationships.

**Figure 2:**
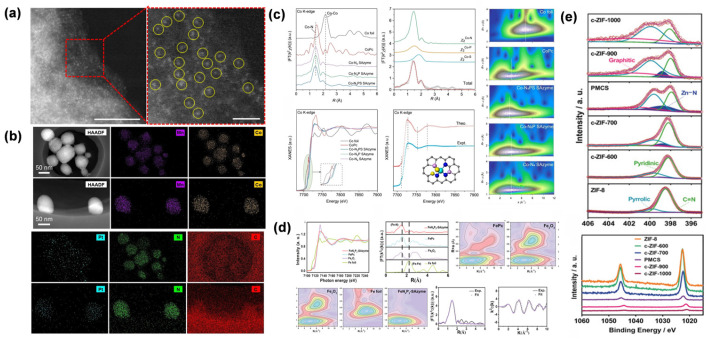
(**a**) AC HAADF-STEM image of FeN_3_P-SAN [55]. Copyright (2021) Spring Nature. (**b**) HAADF-STEM and EDS mapping of MC_2/3_p-NE and MC_2/3_p-SAN [19]. Copyright (2023) John Wiley and Sons. (**c**) Co K-edge EXAFS and XANES analyses of Co-based SANs and reference samples [42]. Copyright (2023) John Wiley and Sons. (**d**) Fe K-edge EXAFS fitting and wavelet transform contour plots of Fe N_4_P_2_-SAN and related references [53]. Copyright (2023) John Wiley and Sons. (**e**) High-resolution XPS spectra of c-ZIF-derived material [28]. Copyright (2024) Elsevier. The combination of sub-angstrom imaging and synchrotron-based spectroscopy provides unambiguous evidence of atomic dispersion and reveals the precise coordination environment of the active centers. For subfigure (**a**), the yellow circles highlight the individual bright dots representing atomically dispersed single atoms, the red frame indicates the selected area for the enlarged view, and the colors represent the corresponding intensity maps. For subfigures (**c**,**d**), the colors in the wavelet transform contour plots represent the signal intensities.

In SAN studies, HAADF-STEM readily visualizes individual metal atoms as isolated, high-contrast dots across the support, providing direct confirmation of single-atom dispersion and demonstrating the absence of clustering seen in conventional nanomaterials. Meanwhile, this technique allows for high-resolution imaging of the carrier’s microstructure, clearly resolving everything from the size distribution of nanoparticles, the pore structure of mesoporous materials, to the layered morphology of two-dimensional materials. Combined with in situ analyses using electron energy loss spectroscopy and energy-dispersive X-ray spectroscopy, HAADF-STEM can not only precisely locate the spatial distribution of single atoms but also simultaneously obtain information on elemental composition, valence states, and their interfacial interactions with the carrier, thereby offering atomic-resolution structural evidence for uncovering the structure–activity relationships of SANs (Figure 2a). Wang et al. employed HAADF-STEM imaging to examine Pt distribution in both a traditional nanozyme (MC_2/3_Cp-NE) and its single-atom analog (MC_2/3_Cp-SAN), where bright contrast spots verified Pt nanoparticles in MC_2/3_Cp-NE and atomically isolated Pt atoms in MC_2/3_Cp-SAN (Figure 2b) [19].

XAFS was employed to characterize the electronic structure and coordination environment of Co SAN by Chen et al. [42], with EXAFS and XANES analyses confirming the atomic dispersion of Co and revealing the distinct coordination configurations (Co-N_3_PS, Co-N_3_P, and Co-N_4_) through bond length, coordination number, and white-line intensity measurements. Wavelet transform analysis of EXAFS data further validated the absence of Co-Co coordination in all SANs (Figure 2c). Qin et al. employed XAFS to elucidate the atomic coordination and electronic state of Fe in the FeN_4_P_2_-SAN material [53], confirming the single-atom dispersion of Fe with a distinct FeN_4_P_2_ coordination configuration. Wavelet transform analysis of EXAFS data further validated the absence of Fe-Fe bonding, while curve fitting of the EXAFS spectra yielded precise bond lengths and coordination numbers for Fe-N, Fe-P, and Fe-S interactions, providing unambiguous structural evidence for the proposed active center configuration (Figure 2d).

Xu et al. employed XPS to investigate the elemental states and coordination environments of the Zn-based SAN (PMCS). The N 1s spectrum displayed signals corresponding to Zn-N (398.7 eV) and graphitic N (401.2 eV), while the Zn 2p peak showed a 0.4 eV negative shift compared with ZIF-8, indicating enhanced electron density around the Zn sites [54]. XPS analysis further demonstrated that the percentage of Zn-N species in PMCS (16.24%) was significantly higher than in samples pyrolyzed at other temperatures, correlating with its enhanced POD-like activity (Figure 2e).

### 2.3. Structural Characteristics of Single-Atom Nanozymes

**Figure 3:**
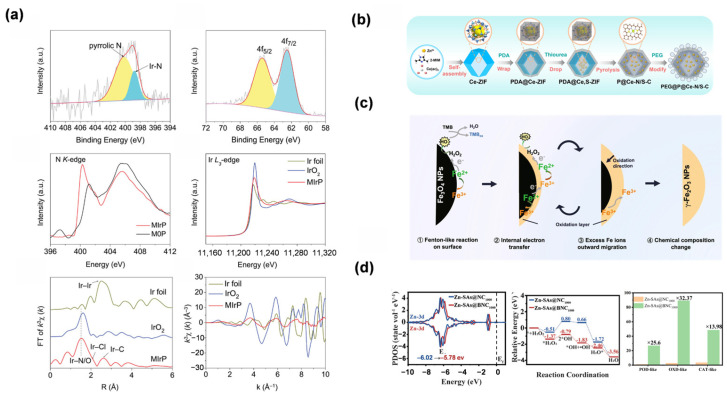
(**a**) Structural and electronic analyses of MIrP, including XPS, XANES, and EXAFS spectra compared with reference samples [56]. Copyright (2023) John Wiley and Sons. (**b**) Synthesis process of PEG@P@Ce-N/S-C [57]. Copyright (2024) American Chemical Society. (**c**) Illustration of the POD-like catalytic mechanism of Fe_3_O_4_ nanoparticles [58]. Copyright (2022) Spring Nature. (**d**) Mechanism and performance analysis of Zn-SAs@BNC1000: Calculated free-energy profiles (**left**), d-band center shifts (**middle**), and the corresponding experimental catalytic efficiency (K_cat_/K_m_) comparison (**right**) during H_2_O_2_ catalysis [59]. Copyright (2024) John Wiley and Sons. These theoretical and experimental results demonstrate that tuning the local coordination and shifting the d-band center effectively lower the activation energy barriers for biomolecule oxidation.

In addition to the characterization methodologies described in Section 2.2, a comprehensive understanding of the intrinsic structural features of SANs is essential. This section focuses on the well-defined coordination environments, local atomic configurations, and unique electronic structures that differentiate single-atom sites from traditional bulk nanomaterials. At the atomic level, SANs immobilize catalytic metal centers as isolated single atoms across the support surface, eliminating the particle aggregation typical of conventional nanoparticles and thereby maximizing atomic efficiency while ensuring a well-defined spatial distribution of active sites [26]. Their metal–carrier interfaces exhibit highly ordered coordination configurations, such as the common planar four-coordinate structure of M-N_4_ (M=Fe, Co, Ni, etc.) or the octahedral configuration of M-O_6_. Such an accurately tuned coordination structure modulates the electron cloud distribution and adjusts the d-orbital characteristics of the metal site, thereby exerting a pronounced impact on substrate binding and the overall catalytic process. Wang and co-workers reported that the active center of the SAN features a planar four-coordinate structure (M-N_4_) formed by noble metal atoms and four N atoms on the porphyrin ring [56]. The existence of this coordination configuration has been confirmed via characterization techniques such as XAFS (Figure 3a). This planar four-coordinate structure mimics the geometric characteristics of the active center of natural PODs, serving as the key structural basis for its efficient enzyme-mimetic catalytic activity.

Furthermore, highly porous supports stabilize single atoms by providing abundant anchoring sites, while strong facet–metal coupling effectively suppresses atomic migration to preserve structural integrity. In the research by Sun et al., the carrier microenvironment synergistically influenced single-atom anchoring through internal coordination regulation and external structural remodeling (Figure 3b). Internally, sulfur atoms were symmetrically embedded in the second coordination sphere of cerium atoms, forming a Ce-N_4_S_2_-C structure. Leveraging the electronegativity and atomic radius characteristics of sulfur atoms, the graphite structure of the carrier was disrupted, generating abundant edge defects and vacancies to enhance the anchoring stability of cerium atoms. Externally, the in situ polymerization of DA maintained the carrier’s three-dimensional framework and enhanced its pore structure, while PEG functionalization improved surface hydrophilicity, thereby promoting better single-atom dispersion and higher metal loading efficiency [57]. The combination of these structural features provides a robust basis for tuning SAN catalytic properties and broadening their analytical applications.

Specifically, the electronic states and local coordination microenvironments of isolated metal centers act as the primary regulators of their redox capacity and substrate affinity. The valence state of metal atoms, as a fundamental property of the active center, directly influences their redox capacity and substrate affinity. For instance, the valence transition between Fe^2+^ and Fe^3+^ is crucial for driving electron transfer in POD-mimicking reactions. Dong et al. revealed that during the POD-mimicking reaction of Fe_3_O_4_ nanozymes, surface Fe^2+^ undergoes oxidation to Fe^3+^ through a Fenton-type process. Simultaneously, electron transfer occurs from the inner Fe^2+^ to the outer layer via Fe^2+^-O-Fe^3+^ bridges, facilitating the regeneration of Fe^2+^ and sustaining the catalytic cycle [58]. However, the outward diffusion of surplus Fe^3+^ served as the kinetic bottleneck of this reaction, and eventually, Fe_3_O_4_ was gradually oxidized to γ-Fe_2_O_3_ as the reaction proceeded, resulting in the depletion of catalytic activity (Figure 3c). Meanwhile, the coordination environment (including coordination number and ligand type) exerted a significant regulatory effect on catalytic activity by altering the local chemical microenvironment of metal atoms. Taking the common M-N_4_ planar four-coordinate structure as an example, the strong electronegativity of N ligands can induce the rearrangement of electron clouds in the d orbitals of metal atoms, reducing the energy barrier of the rate-determining step (RDS) in the reaction.

From an electronic structure standpoint, density functional theory (DFT) studies have revealed that tuning the position of the metal d-band center enables precise modulation of substrate adsorption strength at the active sites. If the d-band center lies close to the Fermi level, reactant adsorption is strengthened while product release becomes more difficult; a modest downward shift in the d-band restores a better adsorption–desorption kinetic balance and thus enhances catalytic performance. Hao et al. [59] demonstrated that B-doping significantly boosted the catalytic efficiency (K_cat_/K_m_) by 13.98–32.37 times, as quantitatively shown in the experimental panel of Figure 3d. This remarkable activity enhancement is theoretically consistent with the lowered reaction energy barriers and the optimized d-band center position. The intrinsic connection between these structural characteristics and catalytic pathways establishes a fundamental basis for the rational design and optimization of SAN performance.

## 3. Activity and Performance Optimization of SANs

**Figure 4:**
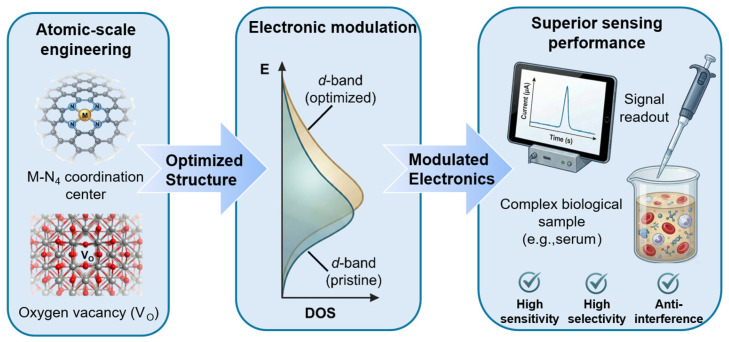
Schematic illustration of the relationship between the structural features of SANs and their sensing performance, highlighting the logic cascade from atomic-scale engineering and electronic modulation to optimized sensing capabilities (high sensitivity, selectivity, and anti-interference) in complex biological matrices. In the atomic-scale engineering models, the grey, red, blue, and yellow spheres represent support metal, oxygen, nitrogen, and active center metal atoms, respectively. In the complex biological sample illustration, the red biconcave discs represent red blood cells, the white textured spheres denote white blood cells, and the smaller blue structures indicate various biomacromolecules and matrix interferents.

Achieving a deep comprehension of the sources of their catalytic activity, investigating approaches to enhance performance, and elucidating distinctions from conventional catalysts are essential for progressing this field from fundamental research toward practical applications [60,61]. By analyzing the metal–carrier synergistic effect and the mechanism of electronic structure regulation, and combining engineering strategies such as carrier design and ligand modification, SANs demonstrate notable improvements in catalytic activity, specificity, and operational stability, bringing new opportunities for breakthroughs in fields like biosensing [62]. To provide a clear visual framework for this transition from structural characteristics to catalytic optimization, Figure 4 illustrates the fundamental link between the atomic-scale engineering of SANs and their resulting sensing performance. As depicted, the precise modulation of the metal coordination environment and the electronic structure—specifically the tuning of the d-band center and the metal–support interaction—serves as the cornerstone for enhancing catalytic activity. By optimizing these atomic configurations, the adsorption kinetics of target biomolecules can be precisely controlled, leading to the superior sensitivity and selectivity observed in biosensing applications.

### 3.1. Activity of Single-Atom Nanozymes

The exceptional catalytic performance of SANs does not stem from the isolated role of metal active centers, but rather from the combined effects of the metal–carrier interface synergistic effect and precise regulation of electronic structure [62,63]. In atomically dispersed systems, the intense coupling between metal atoms and the support modulates the electron density around the active sites, while the unique coordination environment endows them with electronic structure characteristics distinct from traditional nanozymes [64]. In-depth analysis of these action mechanisms constitutes a core scientific issue for revealing the origin of catalytic activity in SANs and achieving rational design of their performance [61].

**Figure 5:**
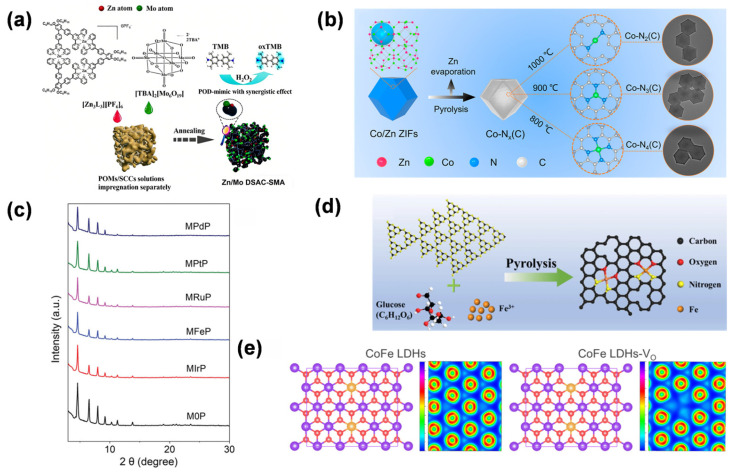
(**a**) Schematic synthesis of Zn/Mo dual SAN confined within a PVA-based aerogel [40]. Copyright (2022) John Wiley and Sons. (**b**) Synthesis of Co-N_x_-SAN [65]. Copyright (2023) American Chemical Society. (**c**) Powder X-ray diffraction pattern for MxP [56]. Copyright (2024) John Wiley and Sons. (**d**) Schematic illustration of the preparation of Fe-CNG by the molecular tailoring strategy [66]. Copyright (2023) Springer Nature. (**e**) DFT calculations elucidating the POD-like mechanism of CoFe LDHs and CoFe LDHs-V_O_ [67]. The color scale bar represents the electron localization function (ELF) values ranging from 0 to 1. The blue regions correspond to ELF = 0, meaning the electrons are completely delocalized or do not exist, while the red regions correspond to ELF = 1, illustrating that the electrons are completely localized. Copyright (2025) American Chemical Society. These optimization strategies demonstrate how tailoring carrier structures and ligand environments can synergistically enhance catalytic activity and selectivity.

As described in Section 2.3, the synergistic interactions between single atoms and supports are pivotal for boosting biosensing performance. In the study by Ma et al. [40], the Zn/Mo dual SAN demonstrated a synergistic effect between Zn and Mo atoms, with their respective coordination configurations (Zn-N_5.6_ and Mo-N_2.9_/O_2.3_) optimizing hydroxyl adsorption energy, enhancing POD-like activity beyond monometallic counterparts. The amphiphilic aerogel substrate, via oxygen-containing groups and 3D porous structure, stabilized high metal loads and facilitates substrate diffusion, reinforcing the metal–support synergy validated by XAFS and DFT (Figure 5a). In the study by Chen et al. [43], the synergistic interaction between Pt single atoms and the N, P, S co-doped hollow carbon support in Pt_TS_-SAN is reflected in the distinctive Pt_1_-N_3_PS coordination, where P acts as an electron donor, and N and S serve as electron acceptors, tuning the electronic structure of Pt, optimizing substrate adsorption, and promoting H_2_O_2_ dissociation, thereby markedly enhancing POD-like activity.

The modulation of d-band electronic configuration via support-driven orbital hybridization effectively governs the substrate adsorption kinetics. By integrating DFT simulations with in situ X-ray absorption spectroscopy analyses, it has been demonstrated that the delocalized π-electron framework of carbon supports, oxygen vacancy sites in metal oxides, and the porous coordination architecture of MOF materials can cooperatively modulate the catalytic reaction pathway via orbital hybridization and electron redistribution, thereby markedly enhancing the catalytic performance of SANs. In the study by Li et al. [65], the OXD-mimicking performance of Co SAN is highly dependent on its local nitrogen coordination. Specifically, the Co-N_3_(C) arrangement demonstrated the highest catalytic efficiency, resulting from favorable O_2_ adsorption, effective production of reactive oxygen species, and a balanced electronic structure, as validated by XAFS characterization and DFT calculations (Figure 5b). In the study by Sun et al. [57], the OXD-mimicking activity of Ce-based SANs was significantly enhanced through precise modulation of their local coordination structure. Specifically, the symmetrical incorporation of sulfur atoms into the secondary coordination shell of Ce generated a Ce-N_4_S_2_-C configuration, which effectively modulated the Ce electronic structure, lowered the energy barrier for O_2_ reduction, and enhanced the orbital coupling between Ce d-orbitals and O p-orbitals, as evidenced by DFT simulations and XAS measurements.

### 3.2. Performance Optimization Strategy

The optimization of SAN performance represents a crucial step in advancing this field from fundamental research to practical applications. Through the precise design of carrier types, ligand structures, and defect engineering, the electronic structure, coordination environment, and surface chemical properties of active centers can be systematically regulated. The distinct characteristics of carriers such as carbon-based materials, metal oxides, and MOFs, the modulation of metal–carrier interfaces by ligand modification, and the active sites introduced via defect engineering collectively form a multi-dimensional strategic system for enhancing the catalytic efficiency, selectivity, and stability of SANs [43,68].

The selection of carrier types stands as one of the core strategies for optimizing the performance of SANs, as the intrinsic properties of different materials significantly influence the dispersibility, electronic structure, and catalytic performance of metal active centers. Diverse supports offer distinct advantages for optimizing SANs performance: Carbon materials utilize high conductivity and π-electron structures to stabilize active centers and modulate d-band electronic states; metal oxides provide abundant anchoring sites via oxygen vacancies and accelerate kinetics through lattice oxygen participation; and MOF-derived architectures enable uniform site dispersion while optimizing substrate diffusion through their highly ordered, designable porous channels. This multidimensional strategic system effectively enhances catalytic efficiency and stability. In the study by Wang et al. [56], the Zr-based MOF carrier, modified by incorporating various noble metal-porphyrin ligands (Ir, Ru, Pt, Pd, and Fe) through a surfactant-assisted strategy with polyvinylpyrrolidone to regulate crystal growth, provided stable coordination environments for metal single atoms, maintained structural integrity as confirmed by powder X-ray diffraction, and enhanced POD-like activity by facilitating substrate adsorption and electron transfer, with MIrP (noble metal-porphyrin-based Ir SAN) exhibiting the highest activity (Figure 5c). In the study by Chu et al. [66], the N-doped graphene nanosheet carrier in Fe-CNG, modified via a molecular tailoring strategy with α-D-glucose as a stabilizer and g-C_3_N_4_ as a precursor, provided abundant N and O coordination sites to anchor Fe single atoms, formed a graphene-like structure with enhanced defect density, and facilitated electron transfer and O_2_ activation, thus synergistically enhancing both the OXD-mimicking and laccase-mimicking catalytic behaviors of the SAN.

As an effective strategy for fine-tuning SANs performance, ligand modification allows for systematic adjustment of metal active centers by introducing heteroatom-containing ligands (e.g., N, P, and S), thereby modulating their local coordination surroundings and electronic configurations. These heteroatoms, by virtue of their unique electron donor/acceptor properties, form directional coordination bonds with metal atoms, significantly altering the geometric configuration of active sites and the distribution of d-orbital electron clouds. For instance, the M-N_4_ planar tetracoordinate structure constructed by N-containing ligands can lower the metal d-band center, optimizing the substrate adsorption–desorption kinetics; while P/S-containing ligands reshape the electron cloud density of metals through strong σ-electron-donating ability, enhancing the affinity for specific substrates. In addition, the steric hindrance effect and electron delocalization characteristics of ligands can precisely regulate the binding mode and reaction pathway of substrates, endowing SANs with excellent substrate specificity and catalytic selectivity. Theoretical calculations and in situ characterizations reveal that ligand modification not only stabilizes the single-atom dispersed state but also regulates the electron transfer at the metal–carrier interface through synergistic effects, providing a structural basis and electronic mechanism for achieving efficient catalysis of SANs. In a study by Wang et al. [69], ligand modification of the SAN via integration of Ir-porphyrin into a Zr-based MOF enhanced its POD-like activity and chemical stability by optimizing the coordination environment (Ir^3+^-N_4_Cl configuration) for stronger H_2_O_2_ adsorption, unique RDS, and lower energy barrier, while surface modification with (−)-epigallocatechin gallate enables targeted delivery to Epstein–Barr Virus-associated nasopharyngeal carcinoma cells for efficient catalytic therapy (Figure 5d).

Defect engineering, as a cutting-edge strategy for optimizing the performance of SANs, provides a unique microenvironment for the construction of active centers by introducing vacancies, edge sites, or lattice distortions on the carrier surface. Atomic vacancies on the carrier surface can serve as high-energy active sites, forming strong interactions with metal atoms through unsaturated coordination bonds. This not only enhances the loading capacity and dispersibility of single atoms but also alters the electronic structure of the metal d-band through local charge rearrangement, thereby optimizing the substrate adsorption energy. Edge sites, due to their low-coordination atoms and unsaturated chemical bonds, can significantly strengthen the electronic coupling between metals and carriers, promoting the activation and transformation of reaction intermediates. Furthermore, crystal disorder arising from lattice distortions generates localized stress fields and defect-related energy levels, which collectively adjust the electron density and coordination patterns surrounding the metal active sites. Such defect-driven structural adjustments not only enrich and expose more catalytic sites but also intrinsically boost the catalytic efficiency of SANs by fine-tuning substrate adsorption–desorption behavior and lowering the activation energy barrier. This provides a novel dimension for the precise regulation and performance enhancement of SANs. In the study by Liu et al. [70], defect engineering was utilized to enhance the catalytic behavior of SAN by converting the coordinatively saturated Cu_1_O_4_ species into unsaturated Cu_1_O_3_ configurations through annealing at 800 °C. These newly formed coordination defects effectively activated isolated Cu centers, promoting the rapid decomposition of H_2_O_2_ to generate hydroxyl radicals (•OH), thereby improving the POD-mimicking performance of the nanozymes. Moreover, these engineered sites imparted superior catalytic stability compared with the conventional oxygen vacancies in CeO_2_-based systems. In the study by Yang et al. [67], defect engineering was exploited to optimize the POD-mimic catalytic performance of CoFe layered double hydroxide quantum dots by introducing oxygen vacancies, which significantly modulated the surface electronic configuration, optimized reactant adsorption energies, reduced reaction energy barriers, and thereby enhanced the generation of •OH from H_2_O_2_ decomposition, as validated by both DFT calculations and experimental results (Figure 5e).

## 4. Applications of Single-Atom Nanozymes in the Detection of Small Biomolecules

SANs, with their unique atomic-scale active centers and tunable catalytic properties, have brought revolutionary breakthroughs to the field of small biomolecule detection [71]. In the directions of disease biomarker screening, neurotransmitter dynamic monitoring, and new sensing technology development, SANs demonstrate remarkable advantages of high sensitivity, high selectivity, and in situ real-time detection, providing innovative approaches and technical support for early disease diagnosis, neural mechanism analysis, and the construction of portable sensing systems [54]. To provide a systematic and critical overview of this field, the research cases discussed in this section are selected based on a rigorous set of criteria, focusing on representative and high-impact studies that demonstrate significant breakthroughs in analytical performance—such as sensitivity, selectivity, and stability—through innovative atomic-scale engineering. Priority is given to work offering profound insights into structure–activity relationships, providing successful validation in complex biological matrices, and showcasing substantial potential for clinical translation. By synthesizing these curated advancements, this section aims to provide a nuanced evaluation of how atomic-level design translates into superior sensing capabilities, transcending mere descriptive reporting.

### 4.1. Glucose

Accurate glucose quantification is fundamental to the management of metabolic disorders, necessitating sensing platforms that combine high sensitivity with exceptional environmental stability [72,73]. SANs have emerged as a sophisticated class of biomimetic catalysts in this field; by precisely engineering the coordination environment of isolated metal sites, SANs can effectively emulate the substrate-specific activation mechanisms of natural glucose oxidase while overcoming the inherent fragility of protein-based enzymes [74]. SANs demonstrate unique molecular recognition and catalytic mechanisms in the field of glucose detection. Their atomically dispersed active centers (such as M-N_4_ and M-O_6_ coordination structures) enable specific adsorption and efficient oxidation of glucose molecules by precisely regulating the metal d-band center and electron cloud density. For example, leveraging their glucose OXD-mimicking behavior, SANs can facilitate the oxidation of glucose by molecular oxygen, producing gluconolactone accompanied by the formation of H_2_O_2_. When leveraging POD-like activity, they further catalyze H_2_O_2_ to participate in colorimetric or electrochemical signal generation. The robust metal–support coupling facilitates efficient electron transfer, which modulates substrate adsorption and desorption dynamics while lowering the reaction energy barrier, thereby markedly improving the overall catalytic performance. This precise molecular-active site electron coupling mechanism, combined with sensing technologies such as fluorescence and electrochemical, enables highly sensitive glucose detection with limits reaching the nanomolar level [75,76].

**Figure 6:**
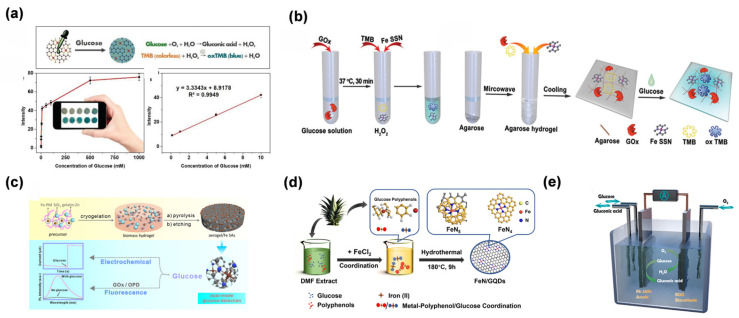
(**a**) Reaction mechanism and visual color response of the type-II glucose biosensing system at various glucose concentrations [77]. Copyright (2019) John Wiley and Sons. (**b**) Schematic of the modified and agarose hydrogel-based colorimetric platforms for glucose detection [78]. Copyright (2020) John Wiley and Sons. (**c**) Preparation route of biomass-derived nitrogen-doped carbon aerogels supporting Fe SAN and enabling dual-mode detection of glucose via fluorescence and electrochemical methods [79]. For subfigure (**c**), the gray porous framework represents the carbon aerogel support; in the atomic model, the brown, blue, and gray spheres represent Fe, N, and C atoms, respectively. Copyright (2021) American Chemical Society. (**d**) Green synthesis of FeN/GQDs nanozyme using *Ananas comosus* leaves as precursors [81]. Copyright (2024) Royal Society of Chemistry. (**e**) Application of the nanozyme for glucose oxidation in a glucose/O_2_ enzymatic biofuel cell [80]. Copyright (2023) American Chemical Society. These studies showcase the transition from paper-based assays to portable hydrogels and self-powered systems, emphasizing the versatility of SANs in clinical glucose monitoring.

Cheng et al. fabricated a carbon nanotube-supported Fe-N-C SAN [77]. This work reported the first application of SAN in paper-based bioassays, enabling colorimetric detection of glucose with limits of detection (LOD) of 0.02 mM. The paper-based platform integrated carbon nanotube/Fe-N-C SAN with colorimetric readouts to enable ultrasensitive detection (Figure 6a). The scientific significance of this work lies in demonstrating that the maximized exposure of Fe-N_x_ moieties can drive ultrasensitive colorimetric responses (LOD: 0.02 mM) on portable substrates, effectively bridging the gap between atomic-level catalysis and point-of-care diagnostics. To further advance portability, Chen et al. fabricated a single-iron-site nanozyme (Fe SAN) supported on porous N-doped carbon (Figure 6b) [78]. An agarose-based hydrogel biosensor was constructed, enabling both colorimetric and quantitative glucose analysis, with a linear range of 0.3–3 mM and a limit of detection of 8.2 nM. The applicability of this method was further confirmed using serum samples. Their work represents the first integration of SANs with hydrogel carriers, advancing the portability of detection platforms. Recent trends have shifted toward multi-functional and sustainable sensing platforms. For instance, Wu et al. reported the fabrication of biomass-derived nitrogen-doped carbon aerogels supporting Fe SAN [79], which enabled dual-mode detection of glucose via fluorescence and electrochemical methods (Figure 6c). This approach highlights the synergistic advantage of multi-signal validation in mitigating false-positive results within complex biological matrices like saliva and sweat. Beyond traditional diagnostics, Zhao et al. presented the rational design of Rh SAN for dual-mode glucose detection via biometabolism and electrometabolism at neutral pH [80]. This work represented a breakthrough by integrating single-atom catalysis with bioenergy conversion, overcoming the fragility and low electron-transfer efficiency of natural enzymes. The dual-mode sensing-energy generation platform paves the way for non-enzymatic glucose monitoring in wearable devices, marking a paradigm shift from traditional diagnostic tools to self-powered biosensing systems (Figure 6e).

Despite these advancements, the transition of SAN-based glucose sensors from laboratory prototypes to routine clinical practice is an ongoing process of optimization. Current research is pivoting toward addressing the intricate matrix effects of biological fluids, such as the non-specific adsorption of proteins, by integrating SANs with sophisticated microenvironment engineering, including protective coatings and molecularly imprinted polymers [82]. Unlike simpler redox molecules, the dynamic range required for blood glucose monitoring (3.9–7.0 mM) necessitates architectures that can maintain high turnover rates without site poisoning [83]. Furthermore, the convergence of machine learning-assisted material design and microfluidic technologies is paving the way for intelligent, real-time, and non-invasive glucose monitoring. These strategic developments are essential for bridging the gap between fundamental research and practical bedside diagnostics, ensuring that the high sensitivity of SANs can be reliably maintained in real-world clinical environments [80,84].

### 4.2. Uric Acid

UA serves as a critical biomarker for metabolic and cardiovascular health, yet its accurate quantification—particularly in electrochemical platforms—is often hampered by the overlapping oxidation potentials of co-existing species like AA. SANs have introduced a refined approach to high-performance UA sensing [85]. By leveraging their atomically dispersed active sites, SANs provide a platform for enhancing molecular recognition and catalytic specificity, effectively mimicking the natural uricase pathway while maintaining superior structural stability [86]. SANs exhibit distinct molecular recognition and catalytic advantages in UA detection, with their detection mechanisms rooted in the mimicry of uricase activity and regulation of electronic structures. By constructing single-atom systems with high catalytic activity (e.g., M-N_4_, M-O_x_ coordination structures), SANs can specifically adsorb UA molecules and efficiently oxidize them into allantoin and H_2_O_2_. The strong metal–support interaction significantly optimizes substrate adsorption–desorption kinetics. By adjusting the position of the metal d-band center, the electron coupling strength between UA and active sites is precisely controlled, thereby enhancing catalytic efficiency and selectivity. The generated H_2_O_2_ can serve as a signaling molecule to enable signal amplification and detection through fluorescence resonance energy transfer, electrochemiluminescence, or colorimetric reactions. For example, colorimetric sensors based on SANs can utilize the reaction of H_2_O_2_ with chromogenic substrates (such as TMB) to visually reflect UA concentration through color changes. Electrochemical detection achieves highly sensitive quantitative analysis of UA by measuring the current signals generated from redox reactions, with detection limits reaching the nanomolar level [71,87].

**Figure 7:**
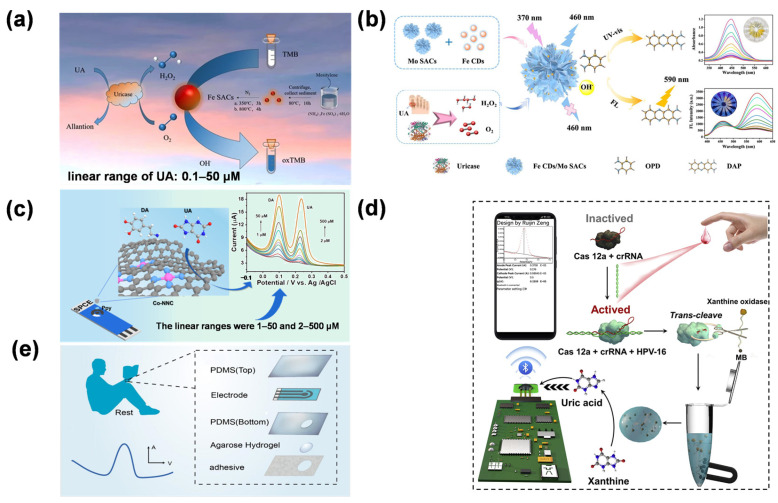
(**a**) Schematic illustration of the colorimetric sensing mechanism of Fe SAN for UA detection [88]. Copyright (2024) Springer Nature. (**b**) Dual-mode detection of H_2_O_2_ and UA [89]. In subfigure (**b**), the different colored lines in the UV-vis spectra represent the response to varying concentrations of UA. Copyright (2024) Elsevier. (**c**) Schematic illustration of Ppy-Co-NNC/SPCE for the detection of UA [91]. Copyright (2024) American Chemical Society. (**d**) Schematic illustration of CRISPR-Cas12a-based target-triggered enzymatic reaction for electrochemical sensing [92]. Copyright (2023) Elsevier. (**e**) Patch placed on the back of the hand to detect UA in sweat without affecting daily work, patch composed of adhesive, hydrogel, polydimethylsiloxane (**bottom**), electrode, and polydimethylsiloxane (**top**) and differential pulse voltammetry signal response that appears when UA is detected [71]. Copyright (2023) American Chemical Society. The integration of single-atom catalysis with CRISPR technology and flexible electronics significantly enhances the selectivity and practicality of UA sensing for personalized medicine.

The evolution of SAN-based UA detection has progressed from fundamental performance validation to the construction of intelligent sensing architectures. Early work by Rui et al. verified the catalytic feasibility of Fe-SANs for colorimetric UA sensing by exploiting their POD-like activity [88] (Figure 7a). Chen et al. described the fabrication of Fe carbon dot (Fe CD)-loaded molybdenum single-atom nanoflowers (Mo SANs) as composite nanozymes [89], enabling dual-mode detection of UA via colorimetry and ratiometric fluorescence (Figure 7b), enabling a linear response to UA ranging from 0.1 to 200 μM (LOD = 0.03 μM). This strategy provides “added value” by overcoming the inherent limitations of single-modal sensing, as the internal self-calibration of optical signals significantly mitigates environmental background interference.

In the electrochemical domain, the focus has shifted toward practical field applications through device integration. Hu et al. achieved an exceptionally broad linear range (0.4–41,950 μM) for UA detection using nitrogen-coordinated cobalt single atoms, demonstrating the robustness of SAN-modified electrodes [90]. Liu et al. described the fabrication of a Ppy-Co-NNC-modified screen-printed carbon electrode (SPCE) for simultaneous electrochemical detection of UA in biological fluids [91]. This Ppy-Co-NNC/SPCE enabled UA detection over a 2–500 μM range (LOD = 0.411 μM) and allowed for dynamic monitoring of UA levels in human sweat during both rest and exercise (Figure 7c). The strategy paves the way for developing advanced biosensing systems for personalized medicine and clinical diagnostics by leveraging the synergistic effects of atomic-level catalysis and flexible electronics. Zeng et al. integrated CRISPR-Cas12a signal amplification with Mo-based SANs to regulate the rate-determining step of UA electrooxidation (Figure 7d). Simultaneously, wearable technologies have been revolutionized by these materials. [92]. Zhang et al. developed a wearable hydrogel patch integrated with Fe SAN for precise UA analysis in resting sweat [71]. The Fe SAN-modified electrode demonstrated high electrocatalytic activity toward UA, enabling a linear detection range of 1–425 μM. A polyaniline-based pH sensor was integrated into the patch to calibrate pH-induced signal variations. Agarose hydrogel with optimized porosity was used to address the low secretion rate of resting sweat, enabling efficient collection and preventing evaporation (Figure 7e). The patch exhibited good biocompatibility and stability, demonstrating its potential for noninvasive, real-time UA monitoring in personalized medicine. Their work bridges single-atom catalysis with wearable technology, representing a breakthrough in clinical-grade POCT for metabolic disease monitoring.

While SANs have demonstrated remarkable efficiency in UA sensing, the selective discrimination of UA from structural analogs in complex biofluids remains a key focus of current research [90,93]. Rather than being a fundamental limitation, the competitive adsorption of species like AA and DA is being addressed through advanced coordination sphere engineering and the construction of multifunctional nano-interfaces that provide selective molecular access [94]. Looking ahead, the synergy between these optimized SAN architectures and smart technologies—such as AI-driven signal processing and microfluidic integration—is expected to yield robust POCT platforms. These developments will not only enhance the reliability of long-term UA monitoring but also accelerate the clinical translation of SANs for real-time metabolic diagnostics [91].

### 4.3. Glutathione

GSH is a central mediator of intracellular redox homeostasis, yet its accurate detection is frequently complicated by the strong affinity of its thiol (-SH) group toward metal surfaces, which typically leads to catalyst deactivation or “poisoning”. To mitigate this effect, the development of SANs with specifically coordinated motifs (e.g., M-N_3_S or M-S_4_) has emerged as a targeted strategy [95]. Utilizing the isolated nature of metal centers and their tunable electronic environments, SANs can transform these thiol–metal interactions into a selective recognition mechanism, facilitating the efficient catalytic oxidation of GSH without compromising catalytic turnover [96]. By designing single-atom systems with M-S_4_ or M-N_3_S coordination motifs (e.g., Au-N_3_S or Fe-S_4_), the strong interactions between metal centers and thiol groups enable selective recognition of GSH. This facilitates the catalytic oxidation of GSH into GSSG dimers, accompanied by either valence changes in the metal centers (e.g., Fe^3+^/Fe^2+^) or electron transfer processes at the carrier interface. The generated oxidation products or electronic signals can be converted through multiple sensing modes: In electrochemical detection, the electron flow released by GSH oxidation catalyzed by SAN-modified electrodes can be quantified by differential pulse voltammetry, with detection limits reaching the nanomolar level. Fluorescence sensing utilizes the fluorescence quenching effect of GSH on the SAN–quantum dot system to achieve visual analysis through fluorescence intensity attenuation. The strong coupling between metal centers and the support material, for example, the delocalized π-electron system in carbon-based supports, can effectively tune the adsorption energy and reaction kinetics of GSH. This modulation markedly enhances detection selectivity and resistance to interference, offering a novel technical strategy for accurate GSH measurement in complex biological environments [39,97].

**Figure 8:**
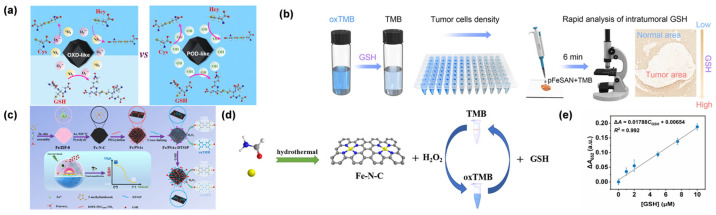
(**a**) Schematic illustration showing selective recognition of GSH over Cys and Hcy via differential POD- and OXD-like activities of Fe-N-C [99]. Copyright (2023) Royal Society of Chemistry. (**b**) Synthesis and sensing performance of porous pFeSAN [95]. Copyright (2023) Springer Nature. (**c**) Fabrication and colorimetric detection of cellular GSH using meshy Fe/PSAs-DTSSP SAN [97]. Copyright (2024) American Chemical Society. (**d**) Colorimetric sensing of H_2_O_2_ and GSH with Fe-N-C SAN [100]. Copyright (2022) Elsevier. (**e**) Calibration curve for GSH quantification [39]. Copyright (2023) MDPI. By tailoring specific coordination motifs, these SANs overcome the interference from similar thiol compounds and enable the visualization of GSH fluctuations in tumor microenvironments. In the molecular and atomic models, the spheres with different colors represent specific atoms: gray for carbon, white for hydrogen, red for oxygen, blue for nitrogen, yellow for sulfur in the biomolecules, and gold/yellow for iron atoms.

Early research focused on establishing the feasibility of single-atom platforms for biothiol sensing. Liu et al. first demonstrated that single-atom Fe-anchored nanodiamonds could achieve nanomolar detection limits for GSH (0.072 μM) by leveraging dual POD- and OXD-like activities [98]. The scientific value of this work lies in proving that atomic dispersion can overcome the signal attenuation typical of bulk nanomaterials in complex matrices. Building on this, Cai et al. reported the preparation of Fe-N-C SAN [99], allowing for the selective detection of GSH over structurally related biothiols such as Cys and homocysteine. (Figure 8a). This approach allowed for the distinction of GSH concentrations between tumor cells (Beta-TC-6) and normal cells (H9C2), revealing elevated GSH levels in the tumor line. Nevertheless, the method exhibited a limited detection range, and its applicability in complex biological samples such as serum has yet to be validated. Their work highlights the potential of tuning SAN dual activities for selective biosensing, though broader dynamic range and biological validation are needed for practical applications. A significant breakthrough in clinical diagnostics was achieved by Chen et al., who developed a biomimetic pFeSAN that enabled rapid (6 min) visualization of GSH in tumor tissues [95]. This development is critical because it integrates single-atom catalysis directly with clinical pathology, overcoming the spatiotemporal limitations of traditional colorimetric assays (Figure 8b). Cai et al. presented a surface engineering strategy to construct a molecular network-bearing Fe-N-C SAN (Fe/PSAs-DTSSP) for highly specific detection of GSH [97], enabling a sensitive colorimetric assay with high selectivity against Cys, homocysteine, and other interferents. Application to A2780 cancer cells showed successful monitoring of GSH fluctuations under glucose deprivation or doxorubicin treatment. Their work addresses the challenge of dynamic GSH monitoring in living cells by integrating single-atom catalysis with responsive surface engineering (Figure 8c). The molecular network strategy overcomes limitations of traditional nanozymes, enabling real-time tracking of GSH in physiological and pathological states. Wei et al. developed Fe-N-C SAN, which conferred both POD- and OXD-like activities (Figure 8d) [100]. In tests using human serum, the approach achieved a GSH recovery rate of 98.7%, confirming its reliability in complex clinical samples. This work validates the practicality of SAN in biomedical applications, providing a non-pyrolytic synthesis route that overcomes agglomeration issues of traditional methods. The Fe-N-C SANs offer a promising platform for POCT, combining high catalytic efficiency with ease of sample handling for clinical diagnostics. Gu et al. employed DFT studies to reveal that the Fe-N_4_ configuration enhances catalytic efficiency by reducing the energy barrier for O-O bond cleavage by 1.2 eV, providing a molecular-level explanation for the high sensitivity of these systems (Figure 8e) [39].

While SANs offer a robust platform for monitoring GSH, achieving unparalleled selectivity in biofluids remains a primary research priority. The persistent challenges posed by the “protein corona” effect and structural thiol analogs are currently being addressed through the development of atomically precise coordination engineering and size-selective porous architectures [99]. For instance, by integrating DFT with mesoporous MOF derivatives, researchers are designing molecular-sieving environments that effectively isolate GSH from interfering biomacromolecules [100]. Looking forward, the synergy between in situ microscopy and machine learning-driven material optimization is expected to elevate the spatiotemporal resolution of SANs, facilitating the transition from laboratory prototypes to high-precision clinical diagnostics at the single-cell level [101].

### 4.4. Ascorbic Acid

AA is an essential regulator of cellular redox homeostasis, yet its precise quantification in physiological environments is frequently hindered by its inherent chemical instability and the presence of structurally similar interferents [102]. To move beyond the limitations of traditional enzyme-based sensors, SANs have emerged as a robust biomimetic platform. By tailoring the coordination environment and metal-support interactions, SANs provide a precise means of modulating the adsorption energy of AA, thereby enhancing the catalytic specificity required for accurate detection in complex biological matrices [103]. SANs exhibit unique catalytic recognition mechanisms in the detection of AA, with their detection principles originating from the efficient regulation of AA oxidation reactions and electronic signal conversion. By constructing single-atom systems with OXD-like activities (such as M-N_4_ and M-O_x_ coordination structures), SANs can specifically adsorb AA molecules through hydrogen bonding or coordination effects, and catalyze their oxidation to dehydro AA. Strong electron coupling at the metal–support interface significantly enhances the charge transfer between AA and the active sites. Computational studies reveal that tuning the d-band center of single-atom sites lowers the activation energy for oxidation reactions, resulting in a 10- to 100-fold increase in catalytic efficiency relative to conventional nanomaterials [104]. The generated oxidation products or electrons released during the reaction can realize signal conversion through multiple sensing modes: In electrochemical detection, the electron flow generated by AA oxidation can be directly measured by cyclic voltammetry or chronoamperometry, with the detection limit reaching the nanomolar level. In colorimetric sensing, SANs catalyze AA to reduce colorless metal ions to form colored products, enabling quantitative analysis through absorbance changes. Lastly, fluorescence strategies utilize the fluorescence quenching or recovery effect of AA on the SAN-fluorescent probe system to construct a highly sensitive detection platform [105].

**Figure 9:**
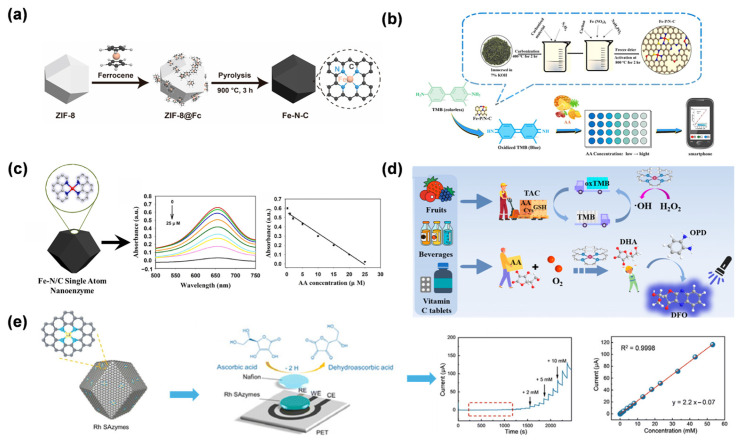
(**a**) Synthesis schematic of Fe-N-C SAN [39]. Copyright (2023) MDPI. (**b**) Smartphone-integrated colorimetric sensor for AA detection based on Fe-P/N-C SAN [107]. The spheres in the structural model denote carbon (brown), nitrogen (blue), oxygen (red), phosphorus (yellow), and iron (orange). The color gradient in the well plate, transitioning from dark blue to light blue/colorless, illustrates the fading of oxidized TMB (oxTMB) with increasing concentrations of ascorbic acid (AA). The red (R), green (G), and blue (B) circles on the smartphone represent the three color channels utilized for digital quantitative analysis. Copyright (2023) Elsevier. (**c**) Structural illustration of Fe-N/C SAN and detection performance [107]. Copyright (2023) Elsevier. (**d**) Optical image and cross-sectional morphology of the screen-printed sensor [105]. Copyright (2023) Royal Society of Chemistry. (**e**) Optical image of the screen-printed sensor and detection performance. The red dashed box in the current-time curve highlights the initial baseline current stabilization period before the successive additions of ascorbic acid. These applications highlight the shift toward portable on-site detection, where electronic modulation via heteroatom doping (e.g., P, N) optimizes the catalytic affinity for AA in food and serum samples.

Gu et al. reported the synthesis of Fe-N-C SAN via pyrolysis of ferrocene-encapsulated ZIF-8 (ZIF-8@Fc) (Figure 9a) [39]. The material features isolated Fe-N_x_ single-atom sites, exhibiting excellent OXD-like activity for catalyzing TMB oxidation to oxTMB, achieving AA detection over 0.1–2 μM with a 0.1 μM detection limit. This study further established a dual-detection platform by exploiting different interaction mechanisms between Fe active centers and target analytes, enabling GSH detection via its inhibitory effect on the nanozyme activity. The strategy not only expands the application of SANs in OXD-mimicking catalysis but also provides a versatile platform for biosensing by harnessing different molecular recognition mechanisms. Li et al. developed Fe-P/N-C SAN using green tea leaves as carbon and nitrogen sources, while NaH_2_PO_2_ was employed as the phosphorus dopant [106]. The P doping modulated the electronic distribution of Fe active sites, enhancing the OXD-like activity of the SAN to catalyze the oxidation of colorless TMB to oxTMB without the need for H_2_O_2_. Leveraging this property, a smartphone-integrated colorimetric sensor was developed for the on-site detection of AA in tropical fruits. The sensor relied on AA’s reducing ability to fade the blue oxTMB, with the color change quantified via the RGB mode of a smartphone camera (Figure 9b). The sensor exhibited a linear detection range of 0.5–100 μM for AA, with a limit of detection of 0.315 μM. By employing P-doping to boost catalytic performance, this approach illustrates how tuning the electronic structure of single-atom active centers can improve portable biosensors, providing an effective and economical strategy for rapid AA detection in food safety applications. Yang et al. proposed a novel colorimetric approach for sensitive AA detection based on an OXD-mimicking Fe-N/C SAN [107]. This nanozyme was prepared by pyrolyzing Fe(acac)@ZIF-8, producing atomically dispersed Fe-N_x_ sites embedded in a porous carbon framework (Figure 9c). Notably, the SAN demonstrated outstanding OXD-like activity, enabling the direct conversion of TMB to blue oxTMB in the absence of H_2_O_2_. The colorimetric assay provided a linear AA detection span of 0.25–25 μM, with a limit of detection of 0.092 μM. Mechanistic investigations indicated that the exceptional catalytic performance arose from the distinctive electronic configuration of single-atom Fe-N_4_ centers, which effectively reduced the energy barrier for TMB oxidation. This approach has been successfully validated in real serum samples, demonstrating its promising applicability in biomedical diagnostics. By integrating single-atom catalysis with colorimetric sensing, this work provides a robust platform for AA analysis in clinical settings. Together, these studies exemplify a progressive research trajectory: from fundamental nanozyme design [39], to catalytic property optimization [106], and finally, to complex biological system integration [107], thereby providing a robust technological pipeline for translating SAN technologies into clinical tools.

Tao et al. performed a bioinspired design of Cu-N/C SAN that mimicked the multi-Cu active sites and redox mechanism of natural AA OXD (AAO) [105]. The Cu-N/C SAN was prepared by pyrolyzing Cu(acac)_2_@ZIF-8, resulting in isolated Cu-N_4_ centers embedded within a nitrogen-doped porous carbon matrix. This structure endowed the material with dual enzyme-mimicking functions, including the AAO-like oxidation of AA and POD-like catalysis for H_2_O_2_-dependent substrate oxidation. The AAO-mimetic activity enabled fluorescent AA detection by catalyzing its transformation to dehydroascorbic acid, which then reacted with o-phenylenediamine to produce a fluorescent signal. The resulting assay exhibited a linear AA response from 10 to 130 μM, with a detection limit of 0.7 μM. At the same time, the POD-like activity enabled colorimetric measurement of total antioxidant capacity by tracking how antioxidants inhibited TMB oxidation. By integrating biomimetic catalysis with dual-sensing modalities, this work establishes a novel paradigm for comprehensive antioxidant capacity assessment, offering high selectivity and practical utility in biomedical and food safety analysis.

Shi et al. described the preparation of Rh SAN through pyrolysis of Rh(acac)_3_@ZIF-8 [108], designed to emulate the active centers of ascorbate POD (Figure 9d). The resulting Rh SAN demonstrated outstanding electrocatalytic performance for AA oxidation, which was ascribed to the presence of atomically dispersed Rh-N_x_ sites within the N-doped carbon matrix. Combined with screen-printing technology, a miniaturized biosensor was constructed. The sensor demonstrated a wide linear range from 10.0 μM to 53.1 mM for AA, with a low detection limit of 0.26 μM. This work establishes a non-enzymatic sensing paradigm by integrating biomimetic single-atom catalysis with portable electronics, providing a robust tool for real-time AA monitoring in clinical and nutritional analysis. The strategy of mimicking natural enzyme active sites with SANs offers new avenues for developing next-generation electrochemical biosensors.

While SANs have established a robust foundation for AA sensing, achieving high-fidelity discrimination within the intricate redox environments of biological fluids remains a pivotal research priority. Rather than viewing the competitive adsorption of structural analogs (e.g., DA and UA) as an inherent limitation, the field is moving toward precision-engineered nano-architectures that provide exclusive molecular access to AA. For instance, the development of MOF-derived porous supports offers a sophisticated means of size-selective filtration, effectively shielding active sites from non-specific protein fouling [108,109]. Furthermore, the synergy between in situ spectroscopic techniques and flexible microelectrode arrays is bridging the gap toward real-time, in vivo monitoring of oxidative stress dynamics [39]. By leveraging machine learning to fine-tune the local electronic environment of single-atom sites, the next generation of SANs will offer the necessary spatiotemporal resolution and analytical precision required for reliable clinical diagnostics in cardiovascular and neurodegenerative pathologies [110].

### 4.5. H_2_O_2_

H_2_O_2_ is a central mediator in cellular redox signaling and a hallmark of oxidative stress, particularly within the tumor microenvironment, where its concentration can deviate significantly from physiological homeostasis [103]. Given its role in driving oncogenic progression and modulating therapy resistance, there is an urgent need for sensing platforms that can provide both high sensitivity and structural robustness in complex biological matrices [42,89,111]. SANs have redefined the landscape of H_2_O_2_ detection by serving as high-performance biomimetic catalysts. By engineering the coordination spheres of isolated metal sites, SANs effectively emulate the active centers of natural peroxidases, allowing for the precise catalytic decomposition of H_2_O_2_ even under the harsh chemical conditions typically found in pathological tissues [112]. The mechanism of H_2_O_2_ detection using SANs is based on their POD-like activity. By constructing single-atom systems with coordination structures such as M-N_4_ and M-O_x_, the metal active centers can efficiently catalyze the decomposition of H_2_O_2_ to generate •OH, which in turn oxidizes chromogenic substrates or triggers signal conversion of fluorescent probes. Take Fe SAN as an example [113]: When interacting with H_2_O_2_, the valence cycle of Fe^2+^/Fe^3+^ drives electron transfer, accelerating the oxidation of TMB to form a blue product [109]. Nanomolar-level detection of H_2_O_2_ can be achieved by quantifying absorbance changes via ultraviolet–visible spectroscopy [42]. In electrochemical systems, electrodes modified with SANs can catalyze the reduction of H_2_O_2_, generating detectable reduction currents. Combined with differential pulse voltammetry, the detection limit can be reduced to the picomolar level [114,115].

**Figure 10:**
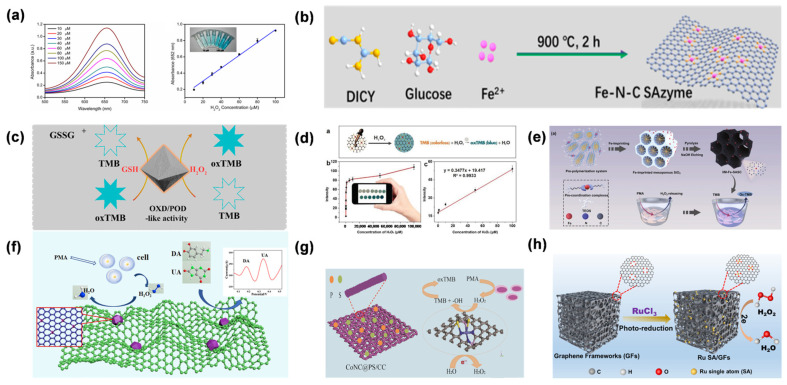
(**a**) UV-vis absorbance spectra and the corresponding linear calibration plot for H_2_O_2_ detection based on Fe SAN [116]. Copyright (2020) Elsevier. (**b**) Schematic illustration of Fe-N-C SAN and detection performance [103]. The spheres with different colors represent different atoms: blue for carbon, red for oxygen, yellow for nitrogen, white for hydrogen, and pink for iron. Copyright (2019) American Chemical Society. (**c**) Schematic presentation for Fe-NDs with POD-like and OXD-like activity [98]. Copyright (2021) Frontiers Media SA. (**d**) Reaction mechanism and visual response for H_2_O_2_ colorimetric detection [77]. a: schematic of the colorimetric reaction mechanism; b: visual response and signal intensity curve across a wide concentration range; c: the corresponding linear calibration plot. Copyright (2019) John Wiley and Sons. (**e**) Synthetic pathway of IIM-Fe-SAN nanoprobes and intracellular H_2_O_2_ sensing [117]. Copyright (2021) Spring Nature. (**f**) Fabrication schematic of Co-N-C and Co-N-C/rGA composites [118]. Copyright (2023) Elsevier. (**g**) Preparation of Co-NC/PS@CC sensor [119]. Copyright (2024) Elsevier. (**h**) Schematic illustration of Ru SA/GFs structural and electrochemical H_2_O_2_ detection [113]. Copyright (2024) American Chemical Society. The high surface area and exposed active sites of these 3D architectures allow for the real-time, in situ tracking of transient H_2_O_2_ secretion from stimulated living cells.

The Zhou group prepared an Fe-SAN via an “isolation–pyrolysis” strategy [116]. In this material, Fe atoms were stably anchored within a nitrogen-doped carbon framework, exhibiting excellent POD-like activity. To validate its catalytic performance, a colorimetric sensing platform for H_2_O_2_ was established. It efficiently catalyzed the H_2_O_2_-mediated oxidation of TMB to produce the blue-colored oxTMB (Figure 10a). The reaction showed high sensitivity toward H_2_O_2_, allowing for quantitative detection over a linear range of 10–150 μM, with a detection limit of 1.8 μM. Building on this foundational work, Jiao et al. [103] reported the preparation of Fe-N-C SAN through high-temperature calcination, employing FeCl_2_, glucose, and dicyandiamide as starting materials (Figure 10b). The as-prepared Fe-N-C SAN exhibited remarkable POD-like activity, enabling sensitive colorimetric detection of H_2_O_2_ with a linear range of 0.5–100 mM and a detection limit of 0.17 μM. Notably, this work demonstrated in situ detection of H_2_O_2_ generated from live HeLa cells using Fe-N-C SAN. The successful intracellular H_2_O_2_ sensing highlights the potential of Fe-N-C SAN in biomedical applications, particularly for real-time monitoring of reactive oxygen species in living cells. Subsequently, to address the limitations in catalytic versatility, Liu et al. described the synthesis of single-atom Fe-NDs through a one-pot in situ reduction approach [98], revealing their distinctive dual-enzyme mimetic behavior, exhibiting both POD- and OXD-like activities. (Figure 10c). The synthesized Fe-NDs facilitated sensitive colorimetric detection of H_2_O_2_ by catalyzing TMB conversion into blue oxTMB in the presence of H_2_O_2_. Their dual-enzyme functionality improved anti-interference performance: the OXD-mimetic activity enabled TMB oxidation independently of H_2_O_2_, whereas the POD-mimetic activity selectively responded to H_2_O_2_, effectively reducing false-positive signals from other interfering substances. This study broadens the use of SANs for monitoring redox metabolites, offering a reliable platform that integrates dual enzyme-like functions for biosensing applications. Further advancing the field toward practical diagnostics, Cheng et al. developed a carbon nanotube-supported Fe-N-C SAN through nanoengineering, achieving a high specific surface area of 1140 m^2^/g and abundant exposure of Fe-N_x_ active sites [77]. The resulting Fe-N-C SAN demonstrated excellent POD-mimicking activity, allowing ultrasensitive paper-based assays for H_2_O_2_, glucose, and AA. The detection limits reached 0.03 μM for H_2_O_2_ (Figure 10d), 0.02 mM for glucose, and 0.03 μM for AA, outperforming many reported nanozyme systems. By integrating SANs with paper-based analysis, this strategy significantly reduces detection costs and enhances field applicability, demonstrating great POCT in resource-limited settings. Most recently, pushing the boundary toward cellular metabolism research, Lyu et al. presented the fabrication of an iron-imprinted single-atom nanozyme (IIM-Fe-SAN) via an ion-imprinting method [117], enabling in situ detection of H_2_O_2_ in MDA-MB-231 cells (Figure 10e). The ion-imprinting process precisely controlled atomic-level dispersion of Fe-N_x_ active sites within a mesoporous carbon matrix, endowing IIM-Fe-SAN with superior POD-like activity (48.5 U/mg) and a detection limit of 23 nM for H_2_O_2_. The nano-probe exhibited excellent selectivity for H_2_O_2_ over biological interferents and allowed quantitative measurement of H_2_O_2_ produced by PMA-stimulated MDA-MB-231 cells, with an average release of 3.48 × 10^7^ molecules per cell. This work validates the utility of ion-imprinted SAN for dynamic monitoring of intracellular H_2_O_2_ in tumor cell metabolism, offering a highly specific tool for biomedical research.

Ding et al. developed a single-atom nanozyme (Fe-SAN/NW) using a zinc-assisted strategy, where heme enzyme-mimicking Fe-N_x_ sites were anchored onto polypyrrole-derived carbon nanowires [115], replicating the pentacoordinate active centers of natural heme enzymes. The Fe-SAN/NW demonstrated a POD-like activity of 42.8 U/mg and facilitated ultrasensitive electrochemical detection of H_2_O_2_, exhibiting a linear range from 5.0 × 10^−10^ M to 0.5 M and a detection limit of 46.35 nM. This represented the first demonstration that structural biomimesis of heme enzymes via atomic-level engineering can significantly enhance catalytic activity. The successful integration of Fe-SAN/NW into electrochemical sensors validates the feasibility of SAN for biomedical diagnostics, laying a foundation for biomimetic design of next-generation catalytic materials. Following the foundational exploration of SANs, Liu et al. presented the fabrication of a 3D hierarchical electrochemical electrode by embedding Co-N-C SAN into reduced graphene oxide aerogels (rGA), denoted as Co-N-C/rGA@GCE [118]. The 3D porous structure of rGA significantly enhanced electron transfer efficiency, enabling ultrasensitive electrochemical detection of H_2_O_2_, DA, and UA (Figure 10f). The constructed sensor achieved a remarkably low H_2_O_2_ detection limit of 0.74 pM over a broad linear range of 3–2991 μM. Utilizing the unique oxidation potentials of DA and UA, the electrode enabled their simultaneous quantitative detection. The strategic integration of Co-N-C nanozymes with rGA not only optimizes the electrocatalytic activity but also extends the application scope to biological fluid analysis. This work highlights the potential of 3D hierarchical nanozyme-rGA composites for multiplexed biosensing in clinical diagnostics and biomedical research. Expanding on this approach, Liu et al. reported the in situ fabrication of P/S co-doped Co-N-C SAN on carbon cloth (Co-NC/PS@CC) through a non-metal doping strategy [119], allowing for real-time detection of H_2_O_2_ released from living cells (Figure 10g). The resulting flexible sensor exhibited a broad linear range of 1–17,328 μM and outstanding stability, owing to the hierarchical porous architecture of the carbon cloth and electronic modulation induced by P/S incorporation. This work validates the feasibility of integrating SAN with flexible substrates for dynamic in situ H_2_O_2_ sensing in living cells, as demonstrated by real-time detection of H_2_O_2_ secretion from MCF-7 cells stimulated by PMA. The strategy of combining atomic-level catalyst design with biocompatible flexible supports paves the way for wearable biosensors in clinical diagnostics. Expanding upon these developments, Jia et al. proposed a strategy to fabricate ruthenium-single-atom-anchored graphene frameworks (Ru SA/GFs) through a one-step photoreduction approach (Figure 10h) [113]. The interconnected porous structure of GFs promoted efficient mass transport and fully exposed single-atom active centers, resulting in the outstanding electrocatalytic performance of Ru SA/GFs toward H_2_O_2_ reduction. Using this material, the sensor achieved an H_2_O_2_ limit of detection of 0.063 μM. This study marks the inaugural use of Ru single atoms for H_2_O_2_ sensing, broadening the scope of single-atom catalysts beyond conventional Fe and Co systems. The superior catalytic performance arises from the tailored electronic configuration and efficient electron transfer of Ru single atoms. These findings not only establish a new paradigm for designing high-performance electrochemical sensors but also highlight the potential of Ru single atoms in biomedical monitoring and beyond.

Despite these analytical milestones, the pursuit of enhanced biocatalytic robustness within the complex tumor microenvironment remains a primary research priority [120]. Unlike simpler detection targets, the thiol-rich environment of biofluids requires architectures that can provide size-selective molecular access. Current efforts are pivoting from simply enhancing sensitivity toward the development of atomically precise, protective architectures, such as MOF-derived molecular sieves that effectively exclude competing biomacromolecules and mitigate the “protein corona” effect [103]. Furthermore, the convergence of SANs with implantable flexible arrays and machine learning-driven models is bridging the gap toward high-fidelity monitoring of H_2_O_2_ fluctuations in vivo [121]. These strategic advancements ensure that the intrinsic activity of SANs can be fully leveraged for precise clinical diagnostics [122,123], facilitating their transition from laboratory prototypes to robust bedside diagnostic tools [42].

### 4.6. Dopamine

DA is a paramount neurotransmitter in the central nervous system, and its precise quantification is essential for the clinical assessment of neurological health. However, the selective monitoring of DA is frequently complicated by the presence of high-concentration interferents like ascorbic acid, which share overlapping oxidation potentials. SANs have introduced a sophisticated biomimetic approach to this challenge; by leveraging their atomically dispersed active centers, SANs provide a tailored platform for the precise regulation of DA’s redox activity [91]. Through the engineering of coordination structures such as M-N_4_ or M-O_x_, these catalysts facilitate the specific adsorption and catalytic oxidation of DA into o-quinone intermediates. The robust interaction between the metal site and its support optimizes the d-band position, markedly accelerating electron transfer and enabling highly sensitive detection—often reaching picomolar limits—via electrochemical or fluorescence-based signal amplification [124,125].

**Figure 11:**
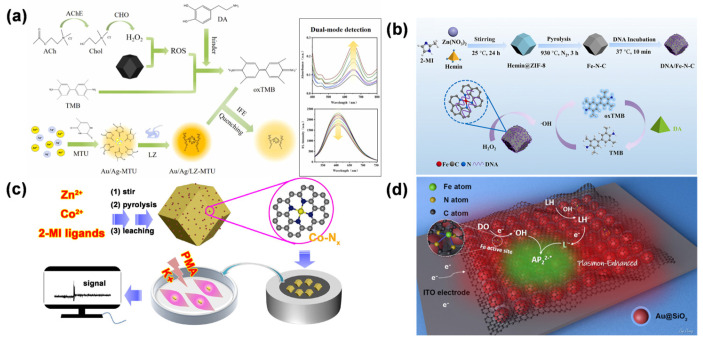
(**a**) Dual-mode sensing platform for DA and AChE detection [125]. Copyright (2025) Elsevier. (**b**) DNA/Fe-N-C synthesis and colorimetric DA detection [126]. Copyright (2023) Royal Society of Chemistry. (**c**) Fabrication of Co-N-C SAN and construction of electrochemical biosensor [127]. Copyright (2021) American Chemical Society. (**d**) Proposed electrochemiluminescence mechanism of Au@SiO_2_-NM/Fe-SAC Luminol-DO system [129]. Copyright (2021) American Chemical Society. The use of DNA modulation and plasmonic enhancement strategies pushes the detection limits of neurotransmitters into the picomolar regime, providing robust tools for neurodegenerative disease research.

Han et al. presented the synthesis of Fe-N_800_ SAN via pyrolysis of Fe-doped ZIF-8 with graphitic carbon nitride (g-C_3_N_4_) [125], enabling the dual-signal detection of DA through a cascade enzymatic reaction. The dual-mode system exhibited sensitivity down to 0.83 μM via fluorescence and 2.7 μM through colorimetric measurement for DA (Figure 11a). This work demonstrates the feasibility of SAN for multiplexed detection in complex biological matrices. The dual-signal strategy mitigates interference and provides orthogonal validation. Wu et al. developed a DNA-modulated Fe-N-C SAN approach to improve its catalytic efficiency [126], enabling highly sensitive colorimetric detection of DA (Figure 11b). The modified system reached a detection limit of 9.56 nM for DA, corresponding to a 436-fold enhancement compared with the unmodified Fe-N-C SAN. This significant improvement resulted from electrostatic interactions between DNA and the substrate. By introducing DNA as a dynamic regulator, the study overcomes the catalytic efficiency limitations of traditional nanozymes, providing a generalizable strategy for constructing highly sensitive biosensors.

Shu et al. reported the synthesis of a Co-N-C SAN via pyrolysis of Co-doped ZIF-8, enabling the ultrasensitive electrochemical detection of DA (Figure 11c) [127], which exhibited a high sensitivity of 979.6 μA/mM/cm^2^, a detection limit of 0.04 μM, and a linear detection range of 0.06–1200 μM for DA. Notably, this work first validated the feasibility of detecting intracellular DA release from PC12 cells. While establishing the foundation for single-atom metal–nitrogen–carbon (M-N-C) materials in electrochemical DA sensing, the study acknowledges limitations in relying solely on single-signal electrochemical detection. Xie et al. reported the synthesis of a single-atom ruthenium biomimetic enzyme (Ru-Ala-C_3_N_4_) for concurrent electrochemical monitoring of DA and UA [128]. The atomically dispersed Ru-Ala-C_3_N_4_ with its tailored electronic structure facilitates efficient electron transfer, producing a pronounced 180 mV separation between DA and UA oxidation peaks. This study marks the first example of a SAN enabling simultaneous monitoring of DA and UA, offering a model for multiplexed sensing in complex biological environments. Bushira et al. presented a novel electrochemiluminescence system by integrating Fe-SAN with two-dimensional plasmonic Au@SiO_2_ nanomembranes [129], enabling ultrasensitive detection of DA (Figure 11d). The developed system enabled DA quantification down to 0.1 nM, exhibiting a linear response across 0.001–1.0 nM. This approach overcomes the sensitivity limitations of traditional electrochemiluminescence systems, pushing DA detection into the picomolar regime. The plasmon-boosted single-atom catalysis strategy provides a general framework for ultrasensitive biomolecule sensing, holding promise for early disease diagnosis by enabling real-time monitoring of trace biomarkers in complex biological matrices. Hu et al. developed the synthesis of a molybdenum SAN (A-Mo-GO) supported on GO, which is further integrated with screen-printing technology to fabricate a flexible electrochemical chip (A-Mo-GO/SPE) for DA detection [130]. The flexible chip maintains structural integrity under bending (radius ≥ 5 mm) and demonstrates excellent stability. The synergy of single-atom catalysis and flexible electronics addresses the portability limitations of traditional detection devices, enabling on-site DA monitoring. This work represents a pivotal advancement in translating SAN to POCT, integrating high-performance catalysis with wearable sensor technology for clinical applications.

While SANs have significantly advanced the sensitivity of DA sensing, achieving robust performance within the intricate neurochemical environment remains an area of active optimization [126]. The challenge of discriminating DA from structurally similar catecholamines (e.g., adrenaline) and other interferents is being addressed by engineering coordination spheres that provide specific molecular recognition through steric hindrance or electronic complementarity. Furthermore, the convergence of these atomically dispersed catalysts with flexible electronics and microfluidic technologies is paving the way for implantable sensors with high spatiotemporal resolution [118]. These strategic advancements in material design and device integration are essential for transitioning SANs from laboratory validation to real-time in vivo monitoring, providing powerful tools for deciphering neural regulation mechanisms and diagnosing neurological disorders.

To systematically evaluate the clinical potential and practical utility of SAN-based sensing platforms, a comprehensive evaluation of their analytical performance—including LOD, linear range, and selectivity—against physiological benchmarks and established gold standard methods is summarized in Table 1. As illustrated, the LODs achieved by these atomically dispersed catalysts for critical biomarkers (e.g., glucose, GSH, and UA) are predominantly within the nanomolar to micromolar range, which effectively covers the spectrum of physiological and pathological concentrations found in human biofluids. Notably, when compared to traditional “gold standard” techniques such as HPLC or enzymatic assays, SAN-based systems demonstrate comparable sensitivity while offering distinct advantages in terms of structural stability, cost-effectiveness, and rapid response times. This comparative analysis highlights that through precise coordination engineering, SANs not only meet the rigorous requirements for real-world diagnostic applications but also provide a robust biomimetic alternative to natural enzymes in complex biological matrices.

## 5. Challenges

Despite the excellent performance of SANs in the detection of small biomolecules, translating laboratory research into practical applications still faces numerous technical bottlenecks. Interference effects in complex biological systems, stability limitations of the materials themselves, and challenges in large-scale preparation processes have become critical factors restricting their further development. To address this, it is urgent to explore systematic solutions through interdisciplinary approaches, spanning from material design and interface engineering to intelligent optimization, so as to overcome the current technical obstacles and promote the industrialization process of SAN-based detection technologies.

## 6. Conclusions and Perspective

In summary, SANs have become a forefront technology for detecting small biomolecules, providing atomically isolated active centers with optimal atomic efficiency and adjustable coordination structures. By carefully engineering the metal-support interfaces and fine-tuning their electronic structures, SANs display enzyme-like catalytic functions with excellent responsiveness, specificity, and durability, allowing quantification of glucose, DA, UA, and other biomarkers down to the picomolar range. The integration of advanced characterization techniques has deepened mechanistic understanding from the atomic to the electronic scale, bridging the gap between material structure and catalytic behavior. Together, these developments provide a strong basis for advancing SAN-based sensing platforms from theoretical studies to real-world diagnostic applications.

Despite these achievements, significant challenges remain before SANs can be widely adopted in clinical and point-of-care settings. The stability of single-atom configurations in complex biological environments, the interference from structurally similar metabolites, and the scalability of synthesis processes continue to limit practical deployment.

Future efforts should emphasize interdisciplinary innovation: coupling SANs with flexible and microfluidic electronics to achieve in vivo, real-time, and multiplexed biosensing; establishing standardized synthetic and evaluation protocols to ensure reproducibility and comparability; and exploring eco-friendly, low-energy fabrication routes to meet sustainability demands. With the aid of computational modeling and intelligent optimization, the rational design of coordination structures and electronic states will accelerate the evolution of SANs from fundamental catalysis research toward intelligent, portable, and clinically relevant biosensing technologies.

## Figures and Tables

**Table 1:** Comparison of analytical performance between SAN-based sensors and clinical gold standard methods for small biomolecule detection.

Analyte	Clinical Level	Standard Method	Material	Mode	LOD	Linear Range	Interferent	Real Sample	Ref.
Glucose	Serum: 3.9–7.0 mM	Hexokinase method	CNT/FeNC	Colorimetry	0.02 mM	0.1–10 mM	AA, UA, DA	Serum	[77]
			Fe SAN	Colorimetry	8.2 nM	0.3–3 mM	AA, UA, DA	Serum	[78]
			NCAG/Fe	Fluorescence	3.1 μM	0.02–1 mM	Fructose, Maltose	Serum	[79]
			FeN/GQDs	Colorimetry	0.36 μM	1–300 μM	AA, UA, DA	Serum	[81]
			Rh_1_ SANs	Electrochemistry	0.5 μM	1 μM–10 mM	AA, UA, DA	Serum	[80]
UA	Serum: 150–420 μM	Uricase-peroxidase method	Fe SANs/N-C	Electrochemistry	-	1–425 μM	AA, Glu, DA	Sweat	[71]
			Fe-N-C SAC	Colorimetry	0.18 μM	0.5–400 μM	AA, UA, DA	Urine	[88]
			Fe-CDs@MoSA-NFs	Electrochemistry	0.03 μM	0.1–200 μM	AA, GSH, DA	Serum	[89]
			A-Co-NG	Electrochemistry	33.3 nM	0.4–1055 μM 1055–41,950 μM	AA, Glu, DA	Serum	[90]
			Ppy-Co-NNC/SPCE	Electrochemistry	0.411 μM	2–500 μM	AA, GSH, L-Cys	Sweat	[91]
			Mo_1_-CN	Electrochemistry	33 nM	1–950 μM	AA, Glu, DA	Not applied	[92]
GSH	Serum: 5–20 μM	Enzymatic recycling	Fe-N-C SAN	Colorimetry	1.3 μM	1–10 μM	Zn^2+^, Ca^2+^, Trp	Serum	[39]
			Fe/PSAs-DTSSP	Colorimetry	0.12 μM	0–80 μM	Glu, Trp, Arg	HeLa cell lysate	[97]
			Fe-NDs	Colorimetry	72 nM	1–25 μM	Glycine, Lysine	Glutathione tablets	[98]
			Fe–N–C SANs	Colorimetry	39.6 nM	0.05–14 μM	L-Cys, AA, Gly	Beta-TC-6 cell lysate	[99]
			pFeSAN	Colorimetry	2.4 nM	50 nM–1 mM	Glu, Trp, Arg	AML12 cell lysate	[95]
			Fe-N-C SANs	Colorimetry	78.33 μM	100–400 μM	L-Cys, Hcy, AA	Beta-TC-6 cell lysate	[100]
AA	Serum: 23–114 μM	HPLC	Fe-N-C SAN	Colorimetry	0.13 μM	1–10 μM	Glu, Trp, Arg	Serum	[39]
			Fe-P/NC SAN	Colorimetry	0.315 μM	0.5–100 μM	Glu, Citric acid	Mango	[106]
			Fe-N/C SAN	Colorimetry	0.092 μM	0.25–25 μM	GSH, L-Cys, UA	Serum	[107]
			Cu-N/C SAN	Colorimetry	0.7 μM	10–130 μM	GSH, L-Cys, UA	Serum	[105]
			Rh-N/C SAN	Colorimetry	0.26 μM	10.0 μM–53.1 mM	GSH, L-Cys, UA	Serum	[108]
H_2_O_2_	Serum: 1–5 μM	Fluorometry	CNT/FeNC	Colorimetry	0.03 μM	0.1–100 μM	Glu, AA, UA	Food	[77]
			Fe-NDs	Colorimetry	0.3 μM	1–60 μM	HAS, BSA, Glu	Glutathione tablets	[98]
			Ru SA/GFs	Electrochemistry	0.063 μM	0.2 μM–32.8 μM 32.8 μM–11.3328 mM	Glu, AA, UA	Serum	[113]
			Fe-SAN/NW	Electrochemistry	46.35 nM	0.5 nM–0.5 M	Glu, AA, UA	Serum	[115]
			Fe SAN	Colorimetry	1.8 μM	10–150 μM	Glu, Fructose, Sucrose	Serum	[116]
			Fe-N-C SAN	Colorimetry	0.17 μM	0.5–100 mM	Glu, Fructose, Glycine	HeLa cells	[103]
			IIM-Fe-SAN	Colorimetry	23 nM	0.25–5 mM	Glu, AA, UA	MDA-MB-231 breast cancer cells	[117]
			Co-N-C	Electrochemistry	0.74 pM	3–2991 μM	Glu, AA, UA	Serum	[118]
			Co-NC/PS@CC	Electrochemistry	0.1687 μM	1–17,328 μM	Glu, AA, UA	Serum	[119]
DA	Serum: <0.2 nM	HPLC-ECD	Fe-N-C SAN	Colorimetry	2.7 μM	5–100 μM	AA, UA, GSH	Serum	[125]
			DNA/Fe-N-C SANs	Colorimetry	9.56 nM	0.01–4 μM 5–100 μM	AA, UA, GSH	Serum	[126]
			Co-N-C-800	Electrochemistry	0.04 μM	0.06–1200 μM	UA, AA, Glu	PC12 cells	[127]
			Ru-Ala-C_3_N_4_	Electrochemistry	0.02 μM	0.06–490 μM	AA, Glu, Fructose	Serum	[128]
			Fe-SANs	Electrochemiluminescence	0.1 nM	0.001–1.0 nM	AA, UA, Glu	Serum	[129]
			A-Mo-GO	Electrochemistry	6.67 nM	0.02 μM–0.97 mM	AA, UA, Glu	Serum	[130]

## Data Availability

No new data were created or analyzed in this study. Data sharing is not applicable to this article.
